# In vitro enzyme inhibitory and cytotoxic studies with *Evolvulus alsinoides* (Linn.) Linn. Leaf extract: a plant from Ayurveda recognized as Dasapushpam for the management of Alzheimer’s disease and diabetes mellitus

**DOI:** 10.1186/s12906-020-02922-7

**Published:** 2020-04-28

**Authors:** Pavithra Mettupalayam Kaliyannan Sundaramoorthy, Kannan Kilavan Packiam

**Affiliations:** Department of Biotechnology, Bannari Amman Institute of Technology, Sathyamangalam, Tamilnadu India

**Keywords:** *Evolvulus alsinoides* (Linn.) Linn., Enzyme inhibition, Alzheimer’s disease, Hyperglycemia, Neuronal disorder, Pearson correlation, Neuroblastoma cell lines, Bioactivity

## Abstract

**Background:**

*Evolvulus alsinoides* (Linn.) Linn. (Convolvulaceae) is a therapeutic herb alleviating brain patterns associated with three categories of regulatory principles of the body, mind, and behaviour. In the current research, enzyme inhibition and cytotoxic potentials of *E. alsinoides* (L.) L. leaf extract has been studied validating its potential application.

**Methods:**

The plant phenolics in the leaf extracts obtained via cold-maceration with solvents viz.: n-hexane, chloroform, ethyl acetate, methanol, and water were quantitatively analyzed. The antioxidant potency was evaluated using 2,2-diphenyl-1-picrylhydrazyl (DPPH) and Ferric Reducing Ability of Plasma (FRAP) assays at five concentrations (100–500 μg). The enzyme inhibition potential was performed with α-amylase, α-glucosidase, and acetylcholinesterase at seven concentrations (25–500 μg). The experiments were done in triplicates and statistically validated using Minitab-17 and SPSS 22.

**Results:**

Water extract contain 45.08 ± 0.02 mg GAE/g, 49.30 ± 0.07 mg GAE/g, 211.21 ± 0.02 mg QE/g tannins, phenolics, flavonoids respectively. Its antioxidant activity was supported by IC_50_ 52.43 ± 0.2 μg/mL (DPPH assay) and 41.58 ± 0.03 (FRAP assay). Methanolic extract inhibits α-amylase with IC_50_ 1.33 ± 0.05 μg/mL. Water extract inhibits α-glucosidase and acetylcholinesterase with IC_50_ 3.58 ± 0.02 μg/mL and 4.46 ± 0.03 μg/mL. Cytotoxicity studies with SH-SY5Y cell-line substantiate the inhibition potential of water extract with IC_50_ 103.0035 μg/mL.

**Discussion and conclusions:**

The extracts with potent antioxidant and enzyme-inhibiting activity were determined. The findings of the research are the first report about the inhibition effects of *Evolvulus alsinoides* (Linn.) Linn extracts against α-amylase, α-glucosidase and acetylcholinesterase. The extracts shall be examined in future studies to evaluate its pharmaceutical potential.

## Background

*Evolvulus alsinoides* (Linn.) Linn. (Convolvulaceae) commonly called as Vishnukranti, is a prostrate perennial herb native of Indian origin from tropical and sub-tropical swampy regions. The plant extracts rejuvenate nervous system, increases sexual power, discharge phlegm and humour from the body, promoting sleep. In Ayurvedic medicine, the whole plant was used as brain tonic and as hepatoprotective [1], amnesia and asthma [2]. Chief phytochemicals in the plant extract are β-sitosterol, scopolin, scopoletin, umbelliferon, triacontane, shankpushpine and betaine. Traditionally local practitioners use this plant extract in the treatment of various neurodegenerative diseases. The earlier reports on therapeutical aspects of the plant encouraged us to carry out the pharmacological activity in this current research. Extraction and phytochemical analysis form the fundamental principle in the isolation of therapeutically active molecules from plant tissues to explore its bioactive potential [3].

Herbal medicines are utilized as traditional and alternative therapy to precisely restore declining cognitive functions. The secondary metabolites present in the medicinal plants possess a wide range of therapeutical properties [4]. The medicinal plant’s aid in retarding the vital metabolic pathways or inhibiting the enzymes is responsible for the biochemical reactions. Moreover, herbal bioactive claims its rewards for their effectiveness, safety and acceptability [5]. Medicinal plants have been regarded as a promising source of lead molecules for new drug development [6].

A neurodegenerative disease that demolishes brain cells leading to distress in thinking, memory, and behaviour is Alzheimer’s Disease (AD) [7]. There is seldom appropriate medication that could restore the normal functions of the brain [8]. To date, no specific treatment for the management of AD has recognized [9]. Acetylcholine Esterase (AChE) are serine hydrolases present in various tissues of the body. AChE terminates signal transmission at cholinergic synapses by hydrolysis of acetylcholine into choline and acetate [3]. AChE possesses a complex molecular polymorphism of quaternary structure in the tissues. The various forms are similar in catalytic activity but differ in hydrophobic interactions and ionic or hydrodynamic parameters [10]. In vitro studies have reported that Acetylcholinesterase (AChE) persuades formation of amyloid fibrils resulting in acetylcholinesterase– beta-amyloid peptide (Aβ) complex that is highly toxic to the brain cells [9]. These complexes get deposited in the brain as amyloid plaques resulting in Alzheimer’s Disease [11]. Amyloid plaques are suitable targets in Alzheimer’s treatment. Acetylcholinesterase (AChE) prevails to be an appropriate target for systematic progress in Alzheimer’s treatment. Treatments with AChE Inhibitors are beneficial in enhancing cognitive malfunction and are most effective in mending perceptive responses [12].

The preliminary studies and epidemiological data proposed that Alzheimer’s Disease as “Type 3 Diabetes”. Earlier studies had reported that correlation between insulin resistance and phosphorylation of tau proteins, heading to the inception and progression of neurodegeneration [13]. Previous studies reported the defects with insulin production and its consequences affecting the central nervous system modulating cognitive function. Reduced insulin signalling and insulin resistance in the brain compete for a substantial role in the pathogenesis of AD. Clinical trials with Humans have proven the efficacy of some oral antidiabetic medications in the improvement of cognition [14].

Diabetes mellitus is a lingering ailment occurred owing to futile utilization of insulin in the body. This condition, called hyperglycemia is a usual risk linked with an increase in the production of free radicals causing tissue injury leading to diabetes. Treatment for diabetes includes control of postprandial hyperglycemia [15]. α-Amylase acts on 1,4-glycosidic linkages in the polysaccharides and converting into disaccharides. α-Glucosidase are enzymes that act upon the disaccharides producing monosaccharides increasing glucose levels in blood [16]. Inhibition of the two enzymes, α-Amylase and α-Glucosidase, becomes a promising approach for diabetes treatment. The inhibition of these enzymes regulates delayed carbohydrate metabolism, decreased glucose retention rate with diminished postprandial plasma glucose level [17]. A wide range of natural and synthetic pharmaceuticals are used to inhibit α-glucosidase and α-amylase [18], thereby reducing blood glucose level aiding in ruling out type 2 diabetes [19]. However, synthetic inhibitors produce adverse effects such as abdominal pain, diabetic ketoacidosis, colonic ulcerations. Hence, nutraceuticals from plants of therapeutic importance are efficient enzyme inhibitors for the management or treatment of metabolic disorders [20].

In the present investigation, neuroprotective and antidiabetic potential of *E. alsinoides* leaf extract was examined. The enzyme inhibition potential of the plant extracts for α-amylase, α-glucosidase and acetylcholinesterase were determined. The current research is the first-time report on the effect of *E. alsinoides* plant extract in inhibiting the viability of neuroblastoma cell lines.

## Methods

### Plant material

*E. alsinoides* (L.) L was collected in a closed paper bag during the flowering season in 2016 from the Western Ghats of Sathyamangalam, Tamilnadu, India and processed in the laboratory. The plant was authenticated at Botanical Survey of India, Coimbatore, Tamil Nadu India [Ref no.: BSI/SRC/5/23/2016/Tech/1711 Dated 27.10.2016]. After authentication, the leaves were removed, washed thoroughly in distilled water, to remove the debris thnen shade dried, powdered and stored in amber bottles [21].

### Preparation of plant extracts

The powdered raw material (20 g) was defatted using pet ether. The defatted sample was successively extracted with 250 mL n-hexane, chloroform, ethyl acetate, methanol, and water by cold maceration method. The extracts were labelled as H, CH, EA, M, W respectively and used alike in all the assays and results. The fat-rich pet ether fraction labelled as P in all subsequent experiments. The solvent-soluble constituents were extracted using the cold maceration method at room temperature with continuous shaking by periodic replacement of fresh solvent every 24 h [22]. The process was repeated thrice with new solvent. Subsequently, extracts were filtered through double-layered cheesecloth under vacuum and concentrated at reduced pressure at 40 °C using a rotary evaporator (Make: Cyberlab, USA & Model: CR2000) and freeze-dried using Freeze Dryer (Make: Martin Christ – Germany, Model: Alpha 1–2 LD Plus) and stored in an airtight container at 4 °C [23].

### Gas chromatography and mass spectroscopy (GCMS) analysis

The plant extracts (1 mg/ml) were dissolved in the appropriate solvent and subjected to GCMS analysis using GC system (Agilent Technologies 6890 N) coupled with Mass spectroscopy (JEOL). Capillary column fused with silica elutes the biomolecules. The system parameters were injection temperature - 220 °C, initial oven temperature - 50 °C increasing up to 250 °C at a rate of 10 °C per min, carrier gas system - Helium (flow rate: 1 mL/min) and interface temperature - 250 °C. The volatiles was identified by comparing the spectrum with National Institute of Standards and Technology (NIST) Chemical Web book database [12]. Pubchem database (https://pubchem.ncbi.nlm.nih.gov/) was used to determine the name, molecular weight and structure of the phytochemicals in the extract.

### Determination of plant Phenolics

The concentration of Tannins and Total Phenolic Compounds [TPC] in the extracts was determined using the Folin–Ciocalteu method. The concentration of Total Flavonoid Compounds [TFC] was determined through aluminium chloride spectrophotometric methods. Tannic acid was used as a standard for estimating total tannins (Y = 3.814x + 0.019, R^2^ = 0.995) and were expressed as Tannic Acid Equivalents (TAE). Gallic acid was used for constructing the standard curve (Y = 6.720x + 0.036, R^2^ = 0.994) and the results were expressed as mg of gallic acid equivalents/g of extract (GAEs) for the total phenolic compounds. Total Flavonoid Content in the extract were expressed as milligrams quercetin equivalents per gram dry extract (mg QE/g) with the calibration curve, Y = 0.012x - 0.104, R^2^ = 0.995 with standard quercetin [24].

### Antioxidant activities

The functional groups possessed by the plant material determine its antioxidant potential. Usage of a single method to evaluate and correlate the antioxidant activity of the compounds is unreceptive. Two significant radical scavenging assays were performed such as 2,2-diphenyl-1-picrylhydrazyl assay (DPPH) and Ferric Reducing of Antioxidant Power Assay (FRAP). Soluble fractions were prepared in the concentration of 0.025–0.5 mg/mL for the tests. The IC_50_ values for each extract were determined by plotting inhibition percentage and sample concentration.

### DPPH Radical Screening Activity

Standard methods with certain modifications were used for determining free radical scavenging ability of the extracts. Alcoholic DPPH solution was prepared by dissolving DPPH (2.4 mg) in absolute methanol (100 mL). Test sample 5 μL was added to 3995 μL methanolic DPPH and vortexed vigorously. The reaction mixture was kept at room temperature for 30 min in complete darkness. The absorbance was read at 515 nm using a spectrophotometer with butylated hydroxytoluene (BHT) as a positive control [25]. The capability of the plant extracts to scavenge the DPPH radical was calculated, and IC_50_ values were determined for each extract.

### Total antioxidant activity (FRAP assay)

The antioxidant competence of the extracts was evaluated using 2,4,6-tripyridyl-S-triazine (TPTZ) in FeCl_3_·6H_2_O solution. Reduction of colourless Fe^3+^ TPTZ complex to blue-coloured Fe^2+^-tripyridyltriazine at low pH by the presence of electron-donating antioxidants was determined. This reaction was measured by computing the absorbance at 593 nm. The constituents of Ferric Reducing Antioxidant Power (FRAP) reagent are 300 mM acetate buffer, 10 mL TPTZ in 40 mM HCl and 20 mM FeCl_3_.6H_2_O in the ratio of 10:1:1 at 37 °C. For each reaction, Diluted plant sample (5 μL) of was added to 3995 μL fresh FRAP reagent and mixed using a vortex mixer and incubated for 30 min at 37 °C. The absorbance was read at 593 nm with ascorbic acid as standard [26]. The reducing potential of the extracts was measured, and IC_50_ value was determined for each extract.

### Acetylcholinesterase inhibition assay

The Acetylcholinesterase inhibitory capacity of each extract was determined using the Ellman method with necessary modification [27]. Briefly, for each assay, 50 μL of 3 mM 5,5′dithiobis nitro benzoic acid, 50 μL AChE (0.5 mg/mL) enzyme from *Electrophorus electricus* (Type V-S, lyophilized powder, ≥1000 units/mg protein in Tris–HCl buffer (pH = 8.0)), 35 μL Tris HCL (50 mM, pH 8.0) and plant extracts (25–500 μg) was mixed and incubated at 37 °C for 10 min in a micro-titre plate. Addition of 25 μL acetylthiocholineiodide (15 mM) initiates the reaction. The blank solution contains all the reagents except the enzyme. The absorbance was recorded at 412 nm after 10 min incubation at 37 °C. IC_50_ (μg/mL) of the extracts that inhibit the enzyme activity was determined.

### α-Amylase inhibition assay

Caraway-Somogyi method [28] with necessary adjustments was adopted to perform the α-Amylase inhibitory activity of the plant extract. Each extract at different concentration (25–500 μg) was added to 50 μL α-amylase (1 mg/mL) in phosphate buffer (pH = 6.9) in 96-well microtiter plate and incubated at 37 °C for 10 min. Addition of starch solution (50 μL, 0.05%) initiates the reaction. After 10 min of incubation at 37 °C, slower addition of 1 M HCl (25 μL) stops the reaction. One hundred microliter Iodine solution was added to the reaction mix. Correspondingly, the control contains all reaction components except the enzyme. The absorbance of the sample was determined at 630 nm (iMark™ Microplate Reader - catalogue #: 168–1130). The percentage of inhibition for each extract at its varying concentration and IC_50_ (μg/mL) were calculated.

### α-Glucosidase inhibition assay

The inhibitory potency of the extract towards α-glucosidase was determined using maltose as the substrate in a micro-titer plate. Both α-glucosidase and maltose were dissolved in 10 mM phosphate buffer (pH 6.86). Forty microliter extract at the varying concentration (25–500 μg) was prepared using 10 mM phosphate buffer and was mixed with 40 μL α-glucosidase (0.3 U/mL). After incubation for 5 min at 37 °C, 20 μL maltose (2 mM) was added to start the reaction. The reaction mixture was incubated at 37 °C for 15 min and stopped by adding 50 μL methanol. The absorbance of the sample and blank was read at 695 nm. The α-glucosidase inhibitory activity was expressed as IC_50_ (μg/mL) [29].

### Cytotoxicity studies using SH-SY5Y cell line by MTT assay

The human neuroblastoma cell line, SH-SY5Y is known as a relevant cellular model for biochemical investigations on Alzheimer’s disease (AD). SH-SY5Y cells are also known as neurosteroid-producing cells which express the chief steroidogenic enzymes [30]. The cell count of SH-SY5Y, neuroblastoma cell line was adjusted to 1.0 × 10^5^ cells/mL using DMEM medium containing 10% FBS. To each well of a 96 well flat bottom microtitre plate, 100 μL of diluted cell suspension (70–80% confluence) was added. After 24 h, at a sufficient population, the cells were centrifuged, and the pellets were suspended at four concentrations viz. 31.25, 62.5, 125, 250 μg/mL. Then, the plates were incubated for 48 h at 37 °C in a 5% CO_2_ atmosphere for microscopic examination, and the observations were recorded every 24 h. After 48 h, 20 μl of MTT (2 mg/mL) was added to MEM-PR (MEM without phenol red) and shaken gently. The plates were further incubated for 2 h at 37 °C in 5% CO_2_ atmosphere. DMSO (100 μl) was added slowly to the plates and gently shaken to solubilize the formed formazan. Absorbance was read at 540 nm using the microplate reader. Per cent cell viability and concentration of extract required to inhibit cell growth by 50% were obtained from dose-response curve [31].

### Statistical analysis

All the assays were carried out in triplicates, and the results expressed as mean ± standard error mean (SEM). The statistical analysis was performed with one-way ANOVA using Minitab-17 Statistical Software Package. Linear regression analysis was carried out to determine IC_50_ values. Bivariate correlation analysis was done using IBM SPSS Statistics 22.0. Pearson’s correlation coefficient was determined and found to be significant.

## Results

### GCMS analyses

A total of 97 different compounds were identified in the five crude extracts. The biomolecules conferred by the plant extracts were identified with the help of spectrum deposited at the NIST library and were summarized in Table 1.

**Table 1:** Results of GCMS Analysis of *E.alsinoides* (L.) L. leaf extracts

S.No	Solvent	RT	Compound	CAS Registry	Molecular Weight g/mol	Molecular formula	Activities Reported
1.	Hexane	20.22	Tridecanoic acid, methyl ester	1731-88-0	228.376	C_14_H_28_O_2_	Antibacterial and Antifungal
2.		21.8	Phytol	150–86-7	296.539	C_20_H_40_O	Antinociceptive and Antioxidant Activities
3.		26.32	2-(2-ethylhexoxycarbonyl) benzoic acid	4376-20-9	278.348	C_16_H_22_O_4_	Anti-depressant
4.		14.62	2,4-Di-tert-butylphenol	96–76-4	206.323	C_14_H_22_O	Anti-fungal
5.		21.47	13-Hexyloxacyclotridec-10-en-2-one	127,062–51-5	280.445	C_18_H_32_O_2_	Antitumor activity
6.		23.73	(2E)-6-methoxy-2-[(4-methoxyphenyl)methylidene]-3,4-dihydronaphthalen-1-one	87,384–01-8	294.344	C_19_H_18_O_3_	No activity reported
7.	Ethylacetate	14.17	Gamma-Terpineol	586–81-2	C_10_H_18_O	154.253	Aroma
8.		15.28	methyl (2Z,6E)-3,7-dimethyldeca-2,6-dienoate	55,283–14-2	C_13_H_22_O_2_	210.317	No activity reported
9.		17.2	methyl tridecanoate	1731-88-0	C_14_H_28_O_2_	228.376	Anticancer
10.		18.85	methyl (13E,16E)-octadeca-13,16-dienoate	56,846–99-2	C_19_H_34_O_2_	294.479	No activity reported
11.		19.77	2-[(9Z,12Z)-octadeca-9,12-dienoxy]ethanol	17,367–08-7	C_20_H_38_O_2_	310.522	No activity reported
12.		20.92	5,7-dihydroxy-2-(4-methoxyphenyl)chromen-4-one (Acacetin)	480–44-4	C_16_H_12_O_5_	284.267	Inhibits neuronal cell death
13.		22.27	3-[2-(1-methylimidazol-2-yl)sulfanylacetyl]chromen-2-one	–	C_15_H_12_N_2_O_3_S	300.332	No activity reported
14.	Chloroform	12.93	1-methyl-2-prop-2-enylsulfanylbenzene	24,309–31-7	164.266	C_10_H_12_S	Used in olefin polymerization
15.		15.53	Quinoline, 5-nitro, 1 - oxide	7613-19-6	190.158	C_9_H_6_N_2_O_3_	Anti-inflammatory
16.		16.15	8,8-Dimethyl-3,3a,4,5,6,7,8,8b-octahydro-2H-indeno[1,2-b]furan-2-one	–	206.281	C_13_H_18_O_2_	No activity reported
17.		17.45	Cetylic acid	57–10-3	256.43	C_16_H_32_O_2_	Biosynthesis of lung lecithin
18.		18.45	16-Octadecenoic acid, methyl ester	56,554–49-5	296.495	C_19_H_36_O_2_	No activity reported
19.		21.08	N-[4-(4,4-Diethyl-1,4-dihydro-2H-benzo(d) [1, 32] oxazin-2-yl)-phenyl]-acetamide	626,409 (Pubchem)	324.424	C_20_H_24_N_2_O_2_	No activity reported
20.		24.25	Quinazolin-4(3H)-one, 3-(3-methoxyphenyl)-2-(2-phenylethenyl)-	112,750–80-8	354.4	C_22_H_16_N_2_O_2_	No activity reported
21.		25.38	Benzoic acid, 2,4-dimethoxy-6-methyl-, (8,8-dimethoxy-2-octyl) ester	312,305–56	368.5	C_20_H_32_O_6_	No activity reported
22.		26.33	2,3,16,17-Octadecanetetraone tetraoxime	34,959–24	370.486	C_18_H_34_N_4_O_4_	No activity reported
23.		22.47	Tricosan-2-ol	–	340.636	C_23_H_48_O	No activity reported
24.	Ethanol	18.33	Octadecanoic acid	57–11-4	C_18_H_36_O_2_	284.484	Anti-viral and anti-inflammatory activities
25.		21.57	octadecyl 2-methoxyacetate	–	C_21_H_42_O_3_	342.564	No activity reported
26.		17.55	[2-(4-Ethyl-phenyl)-4-methyl-2H-phthalazin-1-one]	–	–	–	No activity reported
27.		20.18	Nonadecane-2,4-dione	16,577–69-8	C_19_H_36_O_2_	296.495	Antihistamine activity
28.	Methanol	13.43	Phenol,2-propyl-	644–35-9	C_9_H_12_O	136.194	Flavouring agent
29.		14.12	Flavone	525–82-6	222.243	C_15_H_10_O_2_	Anti-inflammatory
30.		14.17	7-methoxy-2-oxochromene-3-carboxylic acid	20,300–59-8	C_11_H_8_O_5_	220.18	Antitumor
31.		16.15	cyclohexadec-5-en-1-one	37,609–25-9	C_16_H_28_O	236.399	Fragrance
32.		17.12	methyl hexadecanoate	112–39-0	C_17_H_34_O_2_	270.457	Laundry and Dishwashing Products
33.		18.85	Methyl oleate	112–62-9	C_19_H_36_O_2_	296.495	Emulsifiers
34.		20.98	Isopropyl stearate	112–10-7	C_21_H_42_O_2_	326.565	Lubricant and solvent in pharmaceutical formulations
35.		22.82	Phenol, 2,6-bis(1,1-dimethylethyl)-4-[(4-hydroxy-3,5-dimethylphenyl)methyl]-	20,690–84-0	C_23_H_32_O_2_	340.507	No activity reported
36.		24.3	(1Z,5Z)-1,6-dihydroxy-1,6-bis(3-methoxyphenyl)hexa-1,5-diene-3,4-dione	–	C_20_H_18_O_6_	354.358	No activity reported
37.	Water	9.23	Phenol – 2 - propyl	644–35-9	C_9_H_12_O	136.19	Food additive
38.		10	2-methyl-5-phenyl-1*H*-pyrazol-3-one	34,347–81-4	C_10_H_10_N_2_O	174.2	Antiviral and Anticancer
39.		14.12	1-naphthalen-1-ylbut-3-en-1-ol	–	C_14_H_14_O	198.26	Drugs for disorders of the nervous system
40.		14.87	1-methoxy-4-[(4-methoxyphenyl)methyl]benzene	726–18-1	C_15_H_16_O_2_	228.29	Anticancer
41.		16.02	methyl 13-methylpentadecanoate	5487-50-3	C_17_H_34_O_2_	270.5	Antimicrobial
42.		16.6	hexadecanoic acid	57–10-3	C_16_H_32_O_2_	256.42	Flavouring Agent
43.		17.6	methyl (*E*)-octadec-10-enoate	13,481–95-3	C_19_H_36_O_2_	296.5	Anticancer
44.		18.25	3-[2-(1-methylimidazol-2-yl)sulfanylacetyl]chromen-2-one	383,152–05-4	C_15_H_12_N_2_O_3_S	300.3	Anticancer
45.		19.05	1-Ethyl-4-[(4-hexylphenyl)ethynyl]benzene	117,923–34-9	C_22_H_26_	290.4	–
46.		20.28	11-methylidenetricosane	51,732–26-4	C_24_H_48_	336.6	Cosmetic Preparation
47.		21.7	(*E*)-octadec-9-enoic acid	112–79-8	C_18_H_34_O_2_	282.5	Food Additive
48.		23.59	4-[(3,5-di*tert*-butyl-4-hydroxyphenyl)methyl]-2,6-dimethylphenol	20,690–84-0	C_23_H_32_O_2_	340.5	–
49.		29.7	4-[[4-[(4-hydroxy-2,6-dimethylphenyl)diazenyl]-2,3-dimethylphenyl]diazenyl]-3,5-dimethylphenol	–	C_24_H_26_N_4_O_2_	402.5	

The most prevailing compounds present in the plant extracts were 2-(2-ethylhexoxycarbonyl) benzoic acid, (2E)-6-methoxy-2-[(4-methoxyphenyl)methylidene]-3,4-dihydronaphthalen-1-one, 8,8-Dimethyl-3,3a,4,5,6,7,8,8b-octahydro-2H-indeno [1,2-b]furan-2-one,3-[2-(1-methylimidazol-2-yl)sulfanylacetyl]chromen-2-one, Phenol,2,6-bis (1,1-dimethylethyl)-4-[(4-hydroxy-3,5-dimethylphenyl)methyl], [4-(7-acetyloxy-5-methoxy-4-oxochromen-2-yl)phenyl] acetate, 4(3H)-Quinazolinone and 3-(4-hydroxyphenyl)-2-(2-phenylethenyl).

### Estimation of plant Phenolics

The Total Phenolic Content (TPC) of all extract*s* was determined in terms of mg of Gallic Acid equivalents per g extract (mg GAE/g), and the results were displayed in (Table 2). The concentration of tannins in the extracts is present in the order as Aqueous>Methanol>Hexane>Ethyl acetate>Chloroform>Pet ether. The total phenolic content is high in aqueous fraction and decreases in subsequent order as Aqueous>Methanol>Ethyl acetate>Hexane>Chloroform>Pet ether. The total flavonoids in the extracts were determined in terms of mg of Quercetin equivalents per g extract (mg QE/g), and the results were given in (Table 2). Highest flavonoid concentration was found to be present in aqueous extract followed by methanol>ethyl acetate>chloroform>hexane>pet ether.

**Table 2:** Tannins, Total Phenolics and Total Flavanoides in the extracts of *E. alsinoides* (Linn.) Linn

S.No.	Fraction	Tannins (mg GAE/g)	Total Phenolics (mg GAE/g)	Total flavanoides (mg QE/g)
1.	P	0.90 ± 0.02	2.04 ± 0.02	4.79 ± 0.02
2.	H	8.92 ± 0.02	9.69 ± 0.02	20.83 ± 0.02
3.	C	7.44 ± 0.01	6.38 ± 0.03	39.38 ± 0.03
4.	EA	8.57 ± 0.01	10.22 ± 0.02	55.03 ± 0.01
5.	M	23.28 ± 0.01	27.64 ± 0.03	62.26 ± 0.01
6.	W	45.08 ± 0.02	49.30 ± 0.07	211.21 ± 0.02

### Radical scavenging activity

In the present investigation, radical scavenging property of *E. alsinoides* extracts was assessed with three independent experiments, and the values were expressed as mean ± SEM. The most potent activity (IC_50_ of 52.43 μg/mL) was observed with aqueous extract, trailed by methanolic extracts (IC_50_ of 61.55 μg/mL).

### Metal reducing power

*E. alsinoides* extracts showed a decreasing potency in FRAP assay as Water> Methanol> Ethyl acetate > Hexane > Chloroform > Pet ether. Table 3 illustrates the results of antioxidant studies using the extracts of *E. alsinoides*.

**Table 3:** Antioxidant studies using the extracts of *Evolvulus alsinoides* (Linn.) Linn

S.No.	Fraction	DPPH assay IC_50_ μg/ml	FRAP assay IC_50_ μg/ml
1.	P	117.45 ± 0.06	115.72 ± 0.03
2.	H	88.21 ± 0.07	88.63 ± 0.02
3.	C	95.85 ± 0.95	96.52 ± 0.04
4.	EA	78.33 ± 2.2	79.92 ± 0.03
5.	M	61.55 ± 0.6	64.16 ± 0.02
6.	W	52.43 ± 0.2	41.58 ± 0.03
7.	Standard^a^	68.49 ± 0.09	71.05 ± 0.02

^a^– Butylated hydroxy toluene for DPPH assay and Ascorbic acid for FRAP assay

### Acetylcholinesterase inhibitory activity

In the present study, anti-Alzheimer’s potential of *E. alsinoides* was determined. The inhibitory potential of aqueous and methanol fractions was more active in inhibiting AChE than the standard anti-Alzheimer’s drug, galantamine. In contrast, the inhibiting property of other extracts was weak towards AChE (Table 4).

**Table 4:** Enzyme inhibitory activity of the extracts of *Evolvulus alsionoides* (Linn.) Linn

S.No.	Extract	α – Amylase IC_50_, μg/ml	α – Glucosidase IC_50_, μg/ml	Acetyl cholinesterase IC_50_, μg/ml
1.	H	12.35 ± 0.2	8.10 ± 0.1	6.61 ± 0.07
2.	C	14.96 ± 0.05	11.19 ± 0.13	7.55 ± 0.08
3.	EA	7.65 ± 0.1	4.82 ± 0.14	6.03 ± 0.03
4.	M	1.33 ± 0.05	3.80 ± 0.09	4.77 ± 0.04
5.	W	1.34 ± 0.08	3.58 ± 0.02	4.46 ± 0.03
6.	Standard^a^	1.32 ± 0.15	6.46 ± 0.1	2.59 ± 0.09

^a^Acarbose was used as the standard drug in α-amylase and α-glucosidase assays, galantamine in acetylcholinesterase

### α-Amylase and α-glucosidase inhibitory activity

**Fig. 1:**
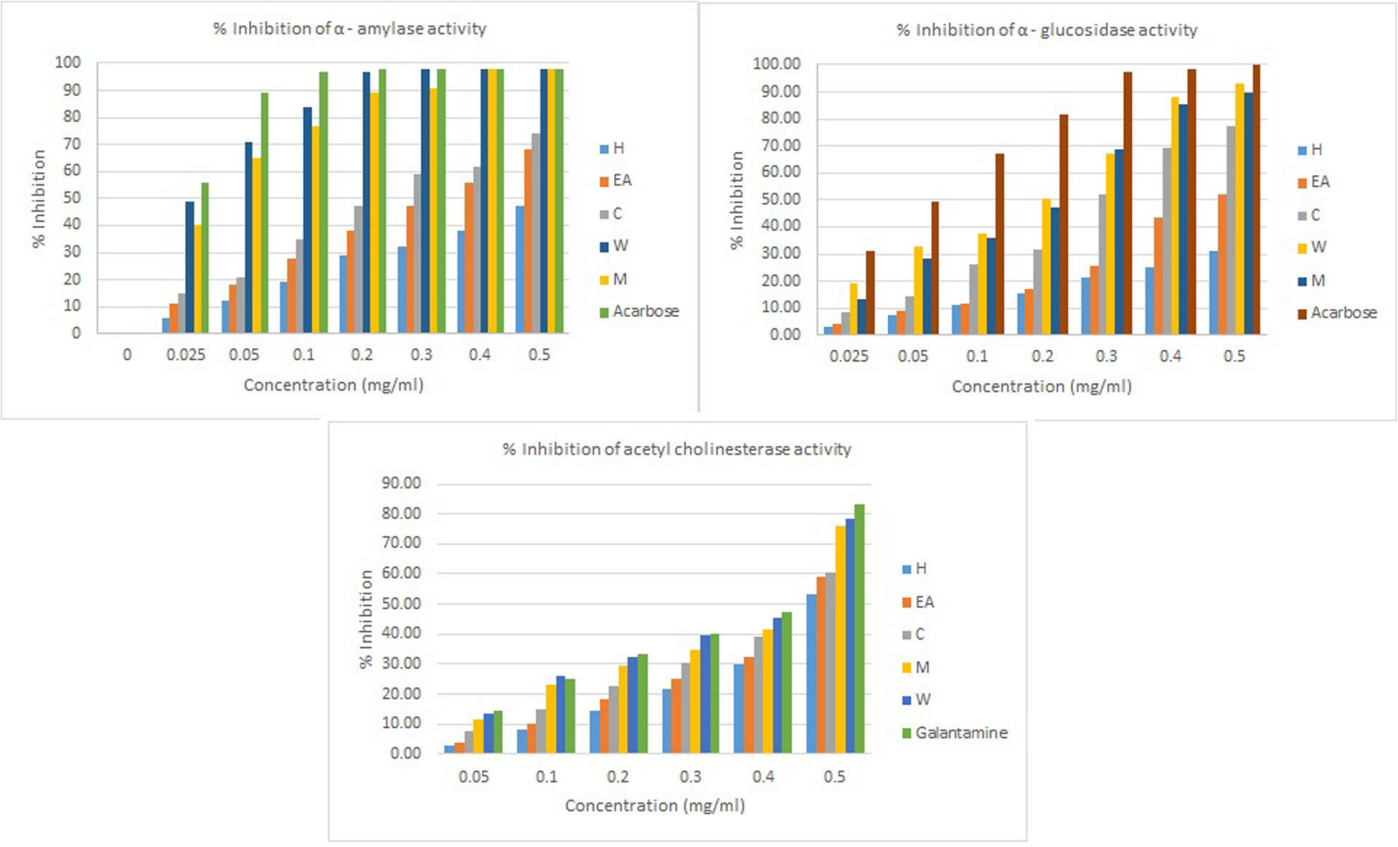
Percentage inhibition of the enzymes by *E.alsinoides* (L.) L. leaf extracts

All the extracts except hexane fraction showed intense α-glucosidase inhibitory activity. Methanol and water extractives showed healthy α-amylase inhibitory activity, whereas chloroform, hexane and ethyl acetate fractions showed weak activity towards α-amylase inhibition (Table 4 and Fig. 1). The antidiabetic drug, Acarbose, with IC_50_ 6.46 mg/mL in the α-glucosidase assay and 1.32 mg/mL in the α-amylase assay was used as a standard.

### Cytotoxicity studies using SH-SY5Y cell line by MTT assay

**Fig. 2:**
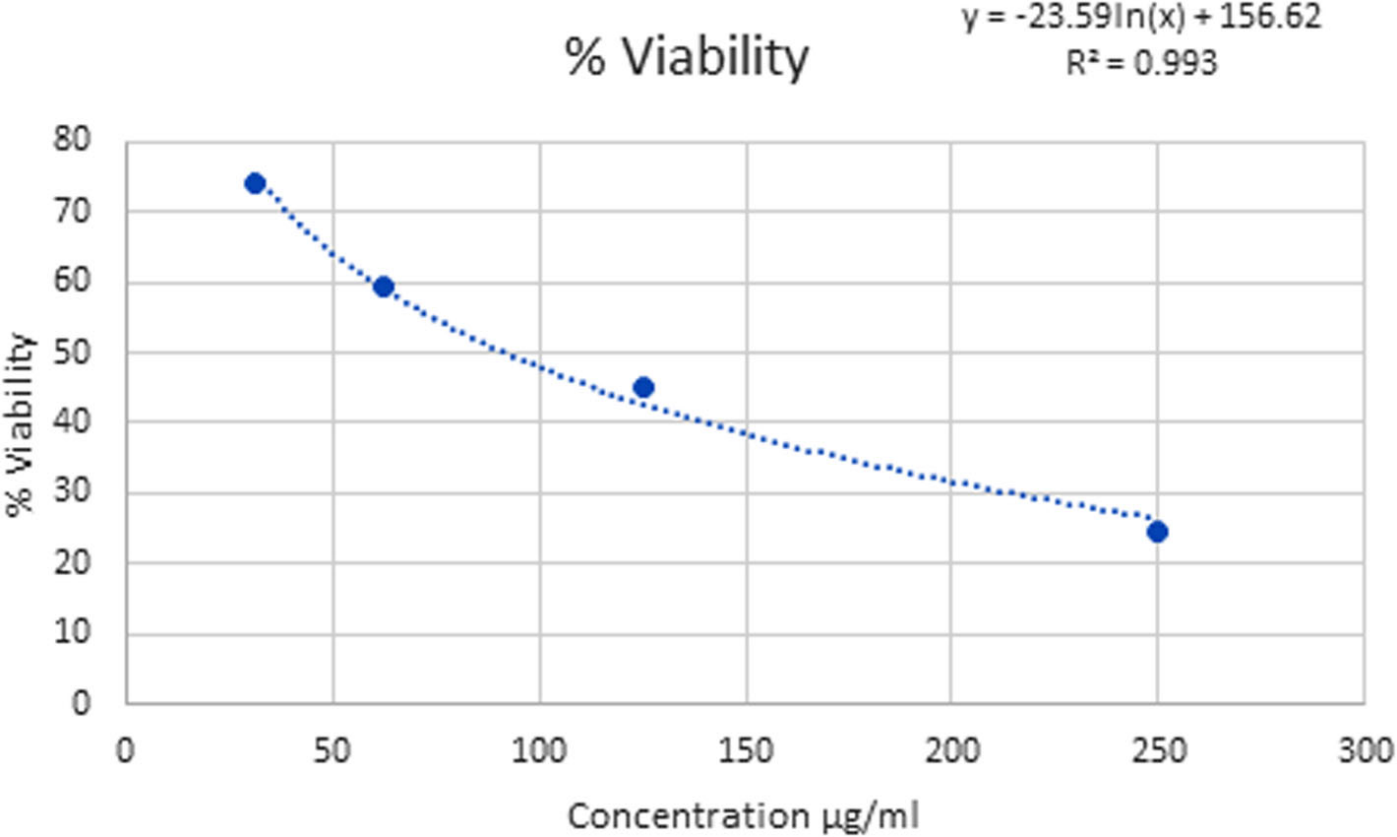
Cytotoxic Study of *E.alsinoides*(L.) L. Leaf extract

The cytotoxic study of *E. alsinoides* (Fig. 2) on SH-SY5Y neuroblastoma cell line using MTT assay showed an IC_50_ value of 103.0035 μg/mL at 70–80% confluency with the percentage viability as 24.7, 45.03, 59.53, 74.36 at 250, 125, 62.5, 31.25 μg/mL.

### Correlation between assays

The statistical relationship between the assays was determined by correlation analysis using SPSS tool. The *p* values resulted from correlation analysis are given in Table 5. The DPPH assay showed a high, positive and significant correlation with α – amylase, α- glucosidase and acetylcholinesterase inhibition (*r* = 0.980, 0.909 and 0.989, *p* < 0.05). Similarly, high, positive and significant correlations were observed between FRAP assay and the enzyme inhibition studies with *r* = 0.920, 0.841 and 0.947 for α – amylase, α- glucosidase and acetylcholinesterase enzymes with *p* < 0.05. A strong positive correlation with *r* = 0.988, *p* < 0.01 is found between alpha-amylase and Acetylcholinesterase inhibitions and a positive correlation with *r* = 0.944, *p* < 0.05 exists between alpha-glucosidase and Acetylcholinesterase inhibitions.

**Table 5:** Pearson’s correlation coefficients of antioxidant activities, α-amylase inhibition, α – glucosidase and acetylcholinesterase inhibition of the extracts

	*DPPH assay*	*FRAP assay*	*α – Amylase*	*α – Glucosidase*	*Acetyl cholinesterase*
*DPPH assay*	1	0.979^a^	0.980^a^	0.909^b^	0.989^a^
*FRAP assay*		1	.920^b^	0.841	0.947^b^
*α – Amylase*			1	0.945^b^	0.988^a^
*α – Glucosidase*				1	0.944^b^
*Acetyl cholinesterase*					1

^a^. Correlation is significant at the 0.01 level (2-tailed)

^b^. Correlation is significant at the 0.05 level (2-tailed)

## Discussion

### GCMS analyses

**Fig. 3:**
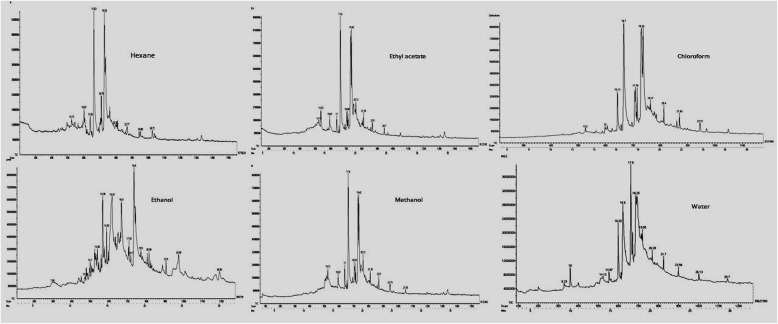
GCMS Chromatogram of organic and alcoholic extracts of *E. alsinoides* (L.) L. leaf

GCMS analysis was carried out to determine the possible chemical constituents from *Evolvulus alsinoides* (L.) L. Analysis of the chromatograms obtained with *E. alsinoides* solvent fractions (Fig. 3) validates the presence of various therapeutically significant phytocompounds. The extracts were found to be rich in aromatic amines, phenols and aromatic phenols that are potent antioxidants in nature.

### Estimation of plant Phenolics

Phenolic acids, flavonoids, and tannins are grouped as plant phenolics. The plant phenolics are regarded to possess potent antioxidant properties that are significant in the prevention of various oxidative stress associated diseases [33]. The results show that the high polar solvents are more useful for extracting antioxidant compounds from plant sources [34]. The polar and aqueous solvents are efficient in the extraction of plant phenolics [35]. The polyphenols have a polarity range between polar and non-polar. Optimum extraction of these aromatic compounds with better efficiency is a result of solvation in the polar solvents due to the interactions between the polar sites of these antioxidant compounds and the solvent [36]. The plant phenolics in the plant sample had polar properties, thus making the polar solvents effective in the extraction. Regression analysis of the obtained results showed a polarity dependent increase with extraction yield, phytochemical content and radical scavenging potency of the extracts [37].

### Radical scavenging activity

The radical scavengers and antioxidant compounds are valuable dietary supplements that protect the human body from various disorders. Radical scavenging assay using DPPH prevails to be a consistent method for determining the antioxidant capacity of natural and synthetic biomolecules. This method is empathetic even with weak antioxidants as DPPH reacts and adequate time specified in the protocol allows DPPH to respond with the free radicals gradually. The technique can be adopted with aqueous and nonpolar organic solvents to examine both hydrophilic and lipophilic antioxidants [38]. The lower value of IC_50_ indicates a higher antioxidant activity [39]. The aqueous extract appears to be more effective than BHT in scavenging DPPH radicals. Shreds of evidence showing a positive correlation between DPPH assay and phenolics have been reported validating the significant contributions of plant phenolics in the DPPH radical scavenging activity [40].

A similar result was shown with *Rhus coriaria* L. fruit extracts, in which higher antioxidant activity was observed with the water extracts than ethanol solubles [41]. The significance of extracting solvent in altering the antioxidant property of wheat bran has been studied recommending high polar solvents for phenolic antioxidants [42]. Studies with *Limnophila aromatica* reported that phenolic acids and flavonoids in the plant extracts were consistent with each other and also their antioxidant activity. Incidence of plant phenolics at prominent levels in the aqueous extract might be owing to the proton-donor and electron-transfer potentialities [43].

### Metal reducing power

The metal-reducing capacity of biomolecules is estimated to be a vital indicator of its antioxidant property. The rate of Fe^3+^ cyanide complex reduction to its ferrous form was measured. The increased, reducing ability of the extracts was determined through the higher absorbance at 700 nm. The results are in correlation with the values obtained through DPPH assay [44]. Due to deficient concentration of phenolics and flavonoids and weak antioxidant activity with DPPH and FRAP, pet ether extract was not used for enzyme inhibition studies. The impact of solvent polarity in influencing the dissolution of antioxidant compounds was ascertained [45].

### Acetylcholinesterase inhibitory activity

The acetylcholinesterases inhibition masters to be a competent approach in Alzheimer’s management [46]. The acetylcholinesterase inhibitors stop the metabolism of acetylcholine [47] increasing neurotransmission [48]. As per the Cholinergic hypothesis, the reduction in acetylcholine (ACh) synthesis leads to AD. Hence, potential therapeutic strategies increase cholinergic levels by inhibiting acetylcholinesterase (AChE) biological activity [49]. Flavonoids claim to be new multipotent leads for AD management owing to significant AChE inhibitory and antioxidant activities [50]. A bioassay-guided fractionation aids in the extraction and isolation of anti-AChE components from the plant extracts [51]. The flavonoids and other phenolic molecules bind to peripheral anionic sites on the Acetylcholinesterase [47]. The polyphenolic compounds with potential AChE inhibitory property and antioxidant activity have been reported [48].

Shankhapushpi is recognized in the Indian system of medicine to improve cognitive function [52]. The results support the use of *E. alsinoides* as a brain tonic in ayurvedic medicine [2]. The secondary metabolites from medicinal plants lessen neuronal dysfunctions by plummeting AChE activity in various regions of the brain [53]. The bioactive that acts on the cholinergic function of the central nervous system are significant in treating Alzheimer’s’ Disease [54].

### α-Amylase and α-glucosidase inhibitory activity

The methanol and water extracts showed the strongest α-amylase and α-glucosidase inhibitory property. Chloroform fraction exhibited week α-amylase and α-glucosidase activities. A positive correlation between antioxidant and antidiabetic properties with the flavonoids content has been reported [55]. A dose-dependent percentage inhibitory activity of the plant extract against alpha-amylase and alpha-glucosidase enzymes was reported.

### Cytotoxicity studies using SH-SY5Y cell line by MTT assay

The results indicate significant dose-dependent inhibition on the growth of SHSY5Y cells with the water extract of *E. alsinoides* leaf. Hence, the water extract of the plant leaves should possess anti-cancerous components that inhibit the growth of SH-SY5Y cell lines. The protective effects of flavonoid-rich extracts support the use of these compounds in the management of oxidative stress-related neurodegenerative diseases such as Alzheimer’s disease and Parkinson’s disease in vitro [36]. The biological activity of these phenolics is consistent with a synergy between them.

### Correlation between assays

The high correlations between the variables support the fact that some set of bioactive in the extracts could be ascribed for its enzyme inhibitory activities [56]. The antioxidant abilities of the plant extract obtained through FRAP assay and DPPH assay were highly correlated signifying that antioxidants in the extracts were capable of scavenging free radicals and reducing oxidants.

Correlation analysis exploring the relationships between the plant phenolics and different antioxidant variables measured in *Halimium halimifolium* reported a linear correlation between antioxidant activity and the plant phenolics [57]. A relatively positive relationship was reported between the phenolics, flavonoids, tannins and their radical scavenging potency in *Rubus* spp. [58]. The plant phenolics possess the ability to scavenge free radicals formed during glycation. Hence DPPH scavenging activity and enzyme inhibition showed a strong positive correlation with anti-oxidant rich extracts. Correlation analysis performed in the present work substantiates that α-amylase, α-glucosidase, and acetylcholinesterase inhibition potential of were attributed with radical scavenging property of the plant extracts.

## Conclusion

Bioactivity-guided screening of biomolecules prevails to be a cost-effective strategy in developing lead molecules for various ailments. From the present research, the leaf extracts of *E. alsinoides* exhibit remarkable inhibitory activity with the methanolic and water extract against α – amylase and α – glucosidase. Also, the methanol and water extracts possess significant acetylcholinesterase activity. Hence leaves of *E. alsinoides* has the potential to be used in ayurvedic decoctions in controlling and treatment of Type II diabetes mellitus and neurodegenerative diseases. The effect of solvent fractions in inhibiting the enzymes and neuroblastoma cell line growth in a dose-dependent fashion was also validated. Furthermore, the results obtained in the current research have opened opportunities for further study in searching novel effective drugs of pharmaceutical importance. Therefore, *E. alsinoides* (L.) L. can be recommended as a plant of significant potential. The current findings expounded that *E. alsinoides* (L.) L., due to its inherent plant phenolics and antioxidant activities, possess enzyme inhibitory properties supporting its traditional application in the management of AD and Diabetes Mellitus. Yet, additional studies with model animals Alzheimer’s disease is required to elucidate the *invivo* efficacy of *E. alsinoides* (L.) L. methodical.

## Data Availability

The datasets analyzed in the study can be made available by the corresponding author upon reasonable request.

## Abbreviations

DPPH: 2,2-diphenyl-1-picrylhydrazyl
FRAP: Ferric Reducing Ability of Plasma
AD: Alzheimer’s Disease
AChE: Acetylcholine Esterase
Aβ: beta-amyloid peptide
TPC: Total Phenolic Compounds
TFC: Total Flavonoid Compounds
BHT: Butylated hydroxytoluene
TPTZ: 2,4,6-tripyridyl-S-triazine
